# Supplementary material for: NetREX-CF integrates incomplete transcription factor data with gene expression to reconstruct gene regulatory networks
Source: Commun Biol. 2022 Nov 23;5:1282. doi: 10.1038/s42003-022-04226-7 (PMC9684490; doi:10.1038/s42003-022-04226-7)
Supplement: Supplementary file 2 — Editorial Assessment Report [file 42003_2022_4226_MOESM2_ESM.pdf]

nature portfolio

GUIDED OPEN ACCESS

nature methods | nature communications | communications biology

Contents of this report

1. **Manuscript details**: overview of your manuscript and the editorial team.
2. **Review synthesis**: summary of the reviewer reports provided by the editors.
3. **Editorial recommendation**: personalized evaluation and recommendation from all 3 journals.
4. **Annotated reviewer comments**: the referee reports with comments from the editors.
5. **Open research evaluation**: advice for adhering to best reproducibility practices.

About the editorial process

Because you selected the **Nature Portfolio Guided Open Access** option, your manuscript was assessed for suitability in three of our titles publishing high-quality work across the spectrum of methods research: ***Nature Methods***, ***Nature Communications***, and ***Communications Biology***. More information about Guided Open Access can be found here.

Collaborative editorial assessment

Your editorial team discussed the manuscript to determine its suitability for the Nature Portfolio Guided OA pilot. Our assessment of your manuscript takes into account several factors, including whether the work meets the **technical standard** of the Nature Portfolio and whether the findings are of **immediate significance** to the readership of at least one of the participating journals in the Nature Portfolio Guided Open Access methods cluster.

Peer review

Experts were asked to evaluate the following aspects of your manuscript:

- **Novelty** in comparison to prior publications;
- **Likely audience** of researchers in terms of broad fields of study and size;
- **Potential impact** of the study on the immediate or wider research field;
- **Evidence** for the claims and whether additional experiments or analyses could feasibly strengthen the evidence;
- **Methodological detail** and whether the manuscript is reproducible as written;
- Appropriateness of the **literature review**.

Editorial evaluation of reviews

Your editorial team discussed the potential suitability of your manuscript for each of the participating journals. They then discussed the revisions necessary in order for the work to be published, keeping each journal's specific editorial criteria in mind.

Journals in the Nature portfolio will support authors wishing to transfer their reviews and (where reviewers agree) the reviewers' identities to journals outside of Springer Nature.
If you have any questions about review portability, please contact our editorial office at guidedoa@nature.com.

nature portfolio

GUIDED OPEN ACCESS
nature methods | nature communications | communications biology

Manuscript details

Tracking number	Submission date	Decision date	Peer review type
GUIDEDOA-21-00236	Aug 23, 2021	Oct 26, 2021	Single-blind

Manuscript title

Reconstruction of gene regulatory networks by integrating a biological model and a recommendation system

Preprint: There is a preprint of this manuscript available at bioRxiv.

Author details

Yijie Wang

Affiliation: Indiana University Bloomington

Editorial assessment team

**Primary editor**	**George Inglis**<br>**Home journal:** *Communications Biology*<br>**ORCID:** 0000-0002-9069-5242<br>**Email:** george.inglis@us.nature.com
**Other editors consulted**	**Lin Tang,** *Nature Methods*, **ORCID:** 0000-0002-6050-0424<br><br>**Doaa Megahed,** *Nature Communications*, **ORCID:** 0000-0002-3455-2992
**About your primary editor**	George received his PhD in Genetics and Molecular Biology from Emory University, where he studied mouse models of voltage-gated sodium channel dysfunction and epilepsy. He also has research experience in epigenomics and in vitro models of neuronal development. George joined the editorial team of *Communications Biology* in September 2020 and is based in the New York office.

Editorial assessment and review synthesis

<table>
<tr><td>Editor's summary and assessment</td><td>Here, the authors present NetREX-CF, a machine learning tool to construct genetic regulatory networks of transcription factors (TFs) and their target genes, with the added benefit of using collaborative filtering to infer networks in the absence of complete knowledge of TF targets (for instance, via ChIP-seq data). They validate NetREX-CF on yeast and Drosophila datasets, showing that it outperforms several alternative tools in identifying putative TF-gene interactions. Importantly, the Drosophila data integrates both existing ChIP-seq and expression profiles, along with RNA-seq following knockdown of 465 TFs in S2 tissue-culture cells.
The editors collectively decided to send this manuscript out to review based on the strength of the benchmarking and value in the new Drosophila transcriptomic datasets. However, there were some concerns regarding the conceptual novelty of this method, in light of several existing alternative approaches, and the limited new biological insight provided in the validation experiments.</td></tr>
<tr><td>Editorial synthesis of reviewer reports</td><td>While the reviewers acknowledge that NetREX-CF outperforms its precursor (NetREX) and that the new Drosophila datasets could be a valuable resource for future studies, they raise several concerns regarding its technical performance relative to existing alternatives, and how it can function in the absence of complete TF binding data. In particular Referee #1 comments on the limited conceptual novelty to alternative methods, and Referees #1-2 highlight the need for further benchmarking or simulations to demonstrate the utility of NetREX-CF. Referees #2-3 also comment on the need for additional data sources (ideally from other organisms), and comment on the limited biological insight provided from the current analysis. Taken together, these concerns prohibit further consideration at Nature Methods and Nature Communications.

However, Communications Biology would be interesting in considering a manuscript that (at a minimum) includes the following revisions:
1. Further discuss the limitations of NetREX-CF and improve benchmarking as outlined by Referees #1-2. While we agree that the manuscript would benefit from the inclusion of datasets from another organism, this point could be addressed as a limitation.
2. Include the simulations suggested by Referee #2, and discuss the applicability of NetREX-CF toward single-cell data.
3. Address Referee #3's concerns regarding the proper use of the yeast and Drosophila networks for validation and testing when inferring genetic regulatory networks.</td></tr>
</table>

Editorial recommendation

Nature Methods<br><br>Revision not invited	Although we and the reviewers appreciate the apparent good performance of the method and the resource value of the new *Drosophila* dataset, unfortunately the paper is not suitable for *Nature Methods* for reasons of methodological novelty (given the availability of many existing approaches) and limited experimental validation of NetREX-CF.
Nature Communications<br><br>Revision not invited	While we and the reviewers appreciate the potential value of the dataset for the *Drosophila* community, without further, extensive validation we find the resource value of this dataset limited. Furthermore, the extent of methodological advance represented by NetREX-CF over the multitude of existing methods is not sufficient for consideration at the journal.
Communications Biology<br><br>Major revisions	As noted by the reviewers, improved benchmarking, model simulations, and discussion of limitations, as well as addressing concerns about appropriate data validation and testing would be necessary for further consideration at *Communications Biology*.

Next steps

**Editorial recommendation 1:**	Our top recommendation is to revise and resubmit your manuscript to *Communications Biology.* We feel the additional experiments required are reasonable within a 6-month time frame.
**Editorial recommendation 2:**	You may also choose to revise and resubmit your manuscript elsewhere. This option might be best if the requested experimental revisions are not possible/feasible at this time.
**Note:**	As stated on the previous page, *Nature Methods* and *Nature Communications* are not inviting a revision at this time. Please keep in mind that the journal will not be able to consider any appeals of their decision through Guided Open Access.

Revision

To follow our recommendation, please upload the revised manuscript files using **the link provided in the decision letter**. Should you need assistance with our manuscript tracking system, please contact Adam Lipkin, our Nature Portfolio Guided OA support specialist, at guidedOA@nature.com.

Revision checklist

- ☐ Cover letter, stating to which journal you are submitting
- ☐ Revised manuscript
- ☐ Point-by-point response to reviews
- ☐ Updated Reporting Summary and Editorial Policy Checklist
- ☐ Supplementary materials (if applicable)

Submission elsewhere

If you choose not to follow our recommendations, you can still take the reviewer reports with you.

Option 1: Transfer to another Nature Portfolio journal
Springer Nature provides authors with the ability to transfer a manuscript within the Nature Portfolio, without the author having to upload the manuscript data again. To use this service, **please follow the transfer link provided in the decision letter.** If no link was provided, please contact guidedOA@nature.com.

> *Note that any decision to opt in to In Review at the original journal is not sent to the receiving journal on transfer. You can opt in to In Review at receiving journals that support this service by choosing to modify your manuscript on transfer.*

Option 2: Portable Peer Review option for submission to a journal outside of Nature Portfolio
If you choose to submit your revised manuscript to a journal at another publisher, we can share the reviews with another journal outside of the Nature Portfolio if requested. You will need to request that the receiving journal office contacts us at guidedOA@nature.com. We have included editorial guidance below in the reviewer reports and open research evaluation to aid in revising the manuscript for publication elsewhere.

Annotated reviewer reports

The editors have included some additional comments on specific points raised by the reviewers below, to clarify requirements for publication in the recommended journal(s). However, please note that all points should be addressed in a revision, even if an editor has not specifically commented on them.

Reviewer #1 information

**Expertise**	This reviewer has expertise in computational biology and genetic regulatory networks.
**Editor's comments**	This reviewer raises important concerns regarding the conceptual novelty of NetREX-CF relative to existing alternative methods, and questions whether it can provide new biological insight. They also stress the need for further benchmarking to better distinguish NetREX-CF from alternative methods.

Reviewer #1 comments

Section	Annotated Reviewer Comments
**Remarks to the Author: Overall significance**	The authors present in the paper a new method NetREX-CF to infer genetic network. NetREX-CF combines various prior information including motif, knockout and ChIP-seq data to predict a gene-TF edge in the network using collaborative filtering (CF). The CF-recommended regulation edge is then corroborated by a sparse NCA-based method. The network edges are predicted through an iterative joint optimization of CF-recommendation and NCA modeling. The authors validated NetREX-CF using yeast network and showed it outperformed several other competing methods. Then they applied it to reconstruct the genetic network in Drosophila Schneider 2 (S2) cell line. They also knocked down all the expressed TFs in S2 cells and generated 1920 RNA-seq data sets. Using this data, they evaluated the performance of the predicted TF-target regulations using NetREX-CF and other methods. The idea of using CF to recommend edges and joint optimization with NCA analysis is interesting and the framework is well articulated in the manuscript. The manuscript falls short on several aspects and the authors should address them before publication.
**Remarks to the Author: Impact**	There are many methods to reconstruct genetic networks and infer important regulators.<br>**This point underscores the need for further benchmarking, but reiterates the concerns regarding conceptual novelty from *Nature Methods* and *Nature Communications*.**
**Remarks to the Author:**	1. The regulatory interactions inferred by NetREX-CF is an average from a collection of gene expression data in different samples rather than in particular cell state or

**Strength of the claims**	sample. Also, the activity of a TF is decided by its expression level but the expression level does not necessarily reflect the activity for many TFs. The authors should discuss whether these issues would limit the applications of NetREX-CF for constructing GRNs in a specific cell type or state.<br>**This point would be necessary for further consideration at *Communications Biology*.**<br><br>2. Inference of GRNs can provide informative insights of regulatory interactions between genes. However, this study falls short of demonstrating the power of NetREX-CF in that it does not showcase what biological insights can be gained by NetREX-CF but not by the other methods. The authors only benchmarked the reconstructed GRN in S2 cells using the extensive knockdown experiments but it is unclear what insights are gained from these analyses.<br>**Please elaborate on how NetREX-CF may enable better discovery of biological insight relative to alternative methods.**<br><br>3. A useful application of GRN is to uncover important regulators in a given cell type at the systems level. Can NetREX-CF outperform the existing methods on this application? This is not discussed in the manuscript.<br>**This benchmarking would be necessary for further consideration at *Communications Biology*.**<br><br>4. Are all the prior data treated equally? How to determine the prior weights for motif, knockout and ChIP-seq data? Are these parameters needed to re-set/re-train for individual applications?<br>**For the sake of reproducibility, please include this information in the Methods or Supplementary Information.**
**Remarks to the Author: Reproducibility**	The source code is available from GitHub.

Reviewer #2 information

**Expertise**	This reviewer has expertise in genetic regulatory networks and machine learning.
**Editor's comments**	This reviewer acknowledges the value of NetREX-CF and its ability to utilize incomplete TF binding data, but stresses the need for further simulations and appropriate validation of the model predictions (see **Major Comment #1**).

Reviewer #2 comments

Section	Annotated Reviewer Comments
**Remarks to the Author: Overall significance**	Reconstructing gene regulatory networks is a challenging but very important task for a better understanding of cellular states in various biological systems. The authors had previously developed a model NetREX that integrates TF binding prior knowledge (e.g., from ChIP-Seq experiments) and gene expression data to learn the regulatory network based on the network component analysis (NCA). In this work, the authors presented a newer version of their tool named NetREX-CF where CF stands for collaborative filtering, which is commonly seen in recommender systems. The CF approach was employed here to address the missing TF binding prior. The authors also presented a solution for the optimization problem of the joint model (CF and NCA). As TF binding prior incompleteness issue is quite common, particularly in those less-studied organisms, the proposed idea is valuable for developing Gene regulatory network reconstruction methods(especially those methods that involve the integrating of TF-gene interaction prior). Nevertheless, I still have several major concerns regarding the NetREX-CF method that the author developed. Please find below the detailed comments.
**Remarks to the Author: Impact**	See the comments in overall significance section
**Remarks to the Author: Strength of the claims**	**Major comments:**<br>1. The biggest concern that I am having is the experimental validation results. I really appreciate the authors' efforts in designing and performing the experiments on Drosophila to validate their model predictions. However, as shown in Figure 4 (middle panel), when predicting the target genes for top-ranked TFs, surprisingly, the random method presents the best performance. This is a bit counter-intuitive. The author did not especially explain the potential reason for such a phenomenon. I wonder whether this is caused by the way that authors used to define the "gold standard" from the experiments (e.g., target genes were defined with $\|log2fc\|>1$, padj<0.05.

Maybe this is too stringent. If we look at table 2, the KnockDown row only has ~50k edges, which is significantly less compared to the edges defined by other TF binding priors. The authors did show that the predicted targets for the top predicted TFs are "more" enriched in functional terms (by GSEA). Although it's making sense that the targets for the same TF should share similar functions, Fig4. (c) is still indirect evidence to support their model predictions.

2. The major advantage of this new model is its ability to deal with missing TF binding priors. The authors should directly demonstrate this point using simulations. For example, the authors could simulate the "TF binding incompleteness" by randomly dropping TF binding priors (say simulate different levels of missing values). By examining the performance of the proposed method on those simulated datasets with incomplete TF binding prior, we can evaluate the robustness of the method and further confirm whether it is good at dealing with missing TF binding priors, as the authors claimed.

This point would be necessary for further consideration at *Communications Biology*.

3. Single-cell data is ever-increasing, and thus the application of the proposed method for GRN inference could be significantly broader if it could be generalized into single-cell data. Although the authors did not mention this specifically in the manuscript, the gene-by-sample expression matrix here could be easily generalized into a gene-by-cell expression matrix for single-cell data. If combining with other single-cell data analysis methods such as Seurat or scdiff, the authors could generate a gene-by-cell expression matrix for each cell population (i.e., cell type or subtype). Then the proposed model could be extended for cell-type-specific GRN reconstruction, which will immensely broaden the application of the proposed NetREX-CF method. I suggest authors add a single-cell section

If feasible, this point should be discussed and integrated into a revised manuscript for further consideration at *Communications Biology*.

4. Also, all the validations/valuations were done in the fruitfly, which might be a bit limited as the GRN is much simpler in fruitfly compared to other more complex organisms such as humans or mice. Therefore, to demonstrate the method's performance in complex organisms, I would suggest authors also perform benchmark analysis following a similar approach as discussed in this 2020 *Nature Methods* paper (https://www.nature.com/articles/s41592-019-0690-6).

While we agree that additional data from humans or mice would improve the impact of the study, this point would not be necessary for further consideration at *Communications Biology*, and could instead be discussed as a limitation.

Minor comments:

1. The Github page provided is NOT publically available. It requires login information. I can't test run the proposed method.

   For the sake of reproducibility, please ensure the GitHub page is readily accessible.

2. On page 10, the first paragraph, the authors mentioned that NetREX-CF achieves the lowest overall average ranking score for all but one. I did not find any other methods that perform better in all 4 tasks.

3. On page 13, "although the performance of NetREX-CFis", it is missing a space between NetREX-CF and is.

4. For the equation on page 6, it shouldn't be -H(S,A) and -F(S,X,Y) as the authors are searching for a solution that minimizes the penalized reconstruction error ||E-SA||^2. I guess " -" is not a sign there. I would suggest either removing it or changing it to another symbol to avoid confusion.

5. On page 1, "this approach becomes the foundation of the current state-of-the-art methods for GRN reconstruction". There are a lot of widely used GRN methods (e.g., GENIE3) that are not based on NCA. A lot of methods are based on regression/correlation. Many others are based on probabilistic graphical models. In each of these categories, we all have good methods that are used widely by the GRN community. It is hard to say which methods are the state-of-the-art ones. I would suggest authors avoid claiming the NCA is the foundation of the current state-of-the-art methods.

6. In the supplement file, the authors are missing a lot of references. There might be other minor issues regarding the supplement. Please take another look and correct all these text/references issues.

Reviewer #3 information

**Expertise**	This reviewer has expertise in computational biology, multi-omic analyses, and genetic regulatory networks.
**Editor's comments**	While this reviewer acknowledges the value of the new *Drosophila* datasets as a resource for the field, they raise concerns about the appropriate evaluation of these experimental datasets. It would be especially critical to address this reviewers' points and justify the current approach for further consideration at *Communications Biology*.

Reviewer #3 comments

**Section**	**Annotated Reviewer Comments**
**Remarks to the Author: Overall significance**	This work reports a new computational method for gene regulatory network (GRN) inference from expression data along with prior knowledge of TF-gene regulatory relationships. It presents a methodological advance over the state-of-the-art, specifically by modifying the authors' previously published NetREX method (*Nature Comm* 2018) to better utilize the prior knowledge. A major contribution of the study is its generation of a large expression data set in *Drosophila* S2 cell lines. Such a data set may be of use to the community in the future.
**Remarks to the Author: Impact**	Despite its positive aspects, the work falls short of convincingly demonstrating the advantage of the new method. Comparisons are made to several existing methods, using one "gold standard" GRN from yeast, but there are concerns about this evaluation. Moreover, the evaluation is on one data set only, out of necessity, since gold standard GRNs are not readily available, but this limitation makes interpretation of perceived improvements difficult. Comparisons are also made using a new "ground truth" constructed using the new data in S2 cells, but these also have significant issues, primarily the fact that the RNAi-based network is dominated by indirect regulatory relationships that are not bona fide GRN edges. In light of this, the work's demonstrable strengths are limited to a technical innovation in the GRN inference methodology and the reporting of a new bulk RNA-seq data set.<br><br>The paper is well written and to-the-point.
**Remarks to the Author: Strength of the claims**	**Major comments:**<br><br>The first main results are shown in Figure 2. Panels A and B compare the accuracy of recovering gold standard GRN edges among different methods.

Overall, NetREX-CF (the presented method) outperforms all competing methods in the per-gene comparison and is about equal to one competing method in the per-TF evaluation. A few comments about this evaluation though: (a) GENIE3 is missing as a competitor. It is a popular method for expression-based GRN inference, even though it does not use prior information. It is a worthwhile comparison to add, to convince readers. (b) The way the results are presented is not entirely satisfactory. It is not clear for example if the “top ranked” genes are systematically biased towards those with fewer regulators (in the gold standard). More commonly, such evaluations are done on a global ranking of all GRN edges, along with ROC and PR curves. Even for per-gene comparison, it may be more meaningful to present per-gene AUROC and AUPRC values as distributions. (c) In the current form, it is not clear if it is useful to have a better predictor at a point on the curve where the average rank of “true TFs” is around 10% or worse. A biologist is unlikely to be interested once the accuracy has dropped to this level, so a performance gap in that range is not particularly helpful.

We would encourage you to compare to GENIE3 as suggested, for further consideration at *Communications Biology*.

Figure 2b suggests that the NetREX-CF method is mostly driven by the prior networks, considering its performance is so closely matched to PriorSum. Is this true?

Figures 2c,d show that the performance advantage of NetREX-CF may be present even on GRN edges not included in prior information. This is promising. The three comments (a,b,c) made above apply here also. Furthermore, it is unclear if the edges present in the gold standard but not in the prior networks are an unbiased and representative “test set”. In my admittedly crude understanding of how the “gold standard” network was constructed originally (and included in the YEASTRACT database), the information represented by the prior networks played an important role, so if an edge is present in the gold standard network but not in the prior networks, it is possible that the edge is actually less reliable. The authors should comment on the possible ascertainment bias introduced in the particular evaluation strategy adopted in Figures 2c,d.

This point would be necessary for further consideration at *Communications Biology*.

Figure 2 is the only systematic evaluation provided in support of the new method. This is because GRN inference evaluation, especially one where prior knowledge is utilized, requires a fairly complete real GRN, and such gold standard networks are simply not available. So the authors understandably rely on the one gold standard network that has been used for evaluation for years now. However, this reviewer believes that such minimal evaluations (on one data set only) do not make a convincing enough case for a new GRN inference method, and perhaps more work is needed in the community to develop

methods for GRN evaluation before methods can be comprehensively assessed and compared. Lacking that, works such as the current work can be considered to have a more limited, technical value.

A substantial contribution of the study is the large set of RNA-seq data measuring the transcriptomic effects of ~500 gene knockdowns, representing "all expressed" TFs in S2 cells in Drosophila. This data set can be of great value for future studies. However, its use as a validation/test set for GRN inference is questionable, despite its comprehensive nature. This is because one expects the majority of edges in the generated "RNAi network" to be indirect regulatory relationships. An examination of the yeast network should indicate to the authors roughly what fraction of genes "downstream" of a TF (i.e., potentially responsive to the TF's knockdown) are indirect targets, and I believe that the authors will find this fraction to be large. Thus, it is hard to read too much into the evaluations of Figure 4. Comments made above in the evaluation of the yeast network apply here too. For instance, if one is interested in counting the target genes for which the "true TFs" are within the top 5-10% (this itself is rather liberal, since it reports ~25-50 TFs, of which presumably a handful are true), NetREX and NetREX-CF have similar performance (Figure 4a, restricted to the left-most part of the curves) and the advantage of the "CF" part, the main innovation in this work, is less clear.

Minor comments:

Grammatical error: "While, Inferelator [3], a method built on network component analysis (NCA), uses given gene expression data and a network prior to estimate TF activity." Please check this sentence.

Typographical error: page 3: "To validate the GRNs bluit by .." Replace bluit with built.

Typo: page 6: "matrix Cij is built form the prior information" … Replace form with from.

Typo: page 14: "defined by each algorithms. we run Gene Set Enrichment Analysis" Replace period with comma.

Typo: page 14: "in this study together whi collected data" … Replace "whi" with "with".

Typo: Acknowledgment to "Alireza Fotuhi Siahpiran" misspells the last name.

Open research evaluation

Data availability

Data availability statement

Please add a Data Availability statement. Please ensure that your Data Availability statement includes accession details for deposited data, mentions where Source data can be found, and states that all other data are available from the corresponding author (or other sources, as applicable) on reasonable request.

More information about our data availability policy can be found here:
https://www.nature.com/nature-portfolio/editorial-policies/reporting-standards#availability-of-data

See here for more information about formatting your Data Availability Statement:
http://www.springernature.com/gp/authors/research-data-policy/data-availability-statements/12330880

Mandatory data deposition

For your RNA sequencing data, submission to a community-endorsed, public repository is mandatory for publication in a Nature Portfolio journal and is best practice for publication in any venue. Accession numbers must be provided in the paper. Examples of appropriate public repositories are listed below:

- Gene Expression Omnibus (Microarray or RNA sequencing data)
- Sequence Read Archive (high-throughput sequence data)
- The European Nucleotide Archive (ENA)

More information on mandatory data deposition policies at the Nature Portfolio can be found at http://www.nature.com/authors/policies/availability.html#data

Please visit https://www.springernature.com/gp/authors/research-data-policy/repositories/12327124 for a list of approved repositories for each mandatory data type.

Other data requests

All source data underlying the graphs and charts presented in the main figures must be made available as Supplementary Data (in Excel or text format) or via a generalist repository (eg, Figshare or Dryad). This is mandatory for publication in a Nature Portfolio journal, but is also best practice for publication in any venue.

The following figures require associated source data: **Fig 3b-f, 4c**

Data citation
Please cite (within the main reference list) any datasets stored in external repositories that are mentioned within their manuscript. For previously published datasets, we ask that you cite both the related research article(s) and the datasets themselves. For more information on how to cite datasets in submitted manuscripts, please see our data availability statements and data citations policy: https://www.nature.com/documents/nr-data-availability-statements-data-citations.pdf<br><br>Citing and referencing data in publications supports reproducible research, by increasing the transparency and provenance tracking of data generated or analysed during research. Citing data formally in reference lists also helps facilitate the tracking of data reuse and may help assign credit for individuals' contributions to research. A number of Springer Nature imprints are signatories of the Joint Declaration on Data Citation Principles, which stress the importance of data resources in scientific communication.
**Code availability and citation**
Please include a statement under the heading "Code Availability", indicating whether and how the custom code/software reported in your study can be accessed, including any restrictions to access. This section should also include information on the versions of any software used, if relevant, and any specific variables or parameters used to generate, test, or process the current dataset. Code availability statements should be provided as a separate section after the Data Availability section.<br><br>Upon publication, Nature Portfolio journals consider it best practice to release custom computer code in a way that allows readers to repeat the published results. Code should be deposited in a DOI-minting repository such as Zenodo, Gigantum or Code Ocean and cited in the reference list following the guidelines described in our policy pages (see link below). Authors are encouraged to manage subsequent code versions and to use a license approved by the open source initiative. Full details about how the code can be accessed and any restrictions must be described in the Code Availability statement.<br><br>See here for more information about our code availability policies: https://www.nature.com/nature-portfolio/editorial-policies/reporting-standards#availability-of-computer-code<br><br>We also provide a Code and Software submission checklist that you may find useful: https://www.nature.com/documents/nr-software-policy.pdf<br><br>Please note: because of advanced features used in this form, you must use Adobe Reader to open the documents and fill it out.

Materials availability

We recommend that any newly generated plasmids are deposited in a community repository (eg, Addgene).

Ethics

Please provide a 'Competing interests' statement using one of the following standard sentences:

1. The authors declare the following competing interests: [specify competing interests]
2. The authors declare no competing interests.

See our competing interests policy for further information: https://www.nature.com/nature-research/editorial-policies/competing-interests

Reporting & reproducibility

Nature Portfolio journals allow unlimited space for Methods. The Methods must contain sufficient detail such that the work could be repeated. It is preferable that all key methods be included in the main manuscript, rather than in the Supplementary Information. Please avoid use of “as described previously” or similar, and instead detail the specific methods used with appropriate attribution.

Nature Portfolio wishes to improve the reproducibility of the work that we publish. Thus, we ask that you present all key equations in the main manuscript rather than in the supplementary information. To improve reproducibility of your analyses, please detail the methods used for data fitting and provide a rationale for this approach.

Please state in the legends how many times each experiment was repeated independently with similar results. This is needed for all experiments, but is particularly important wherever results from representative experiments (such as micrographs) are shown. If space in the legends is limiting, this information can be included in a section titled “Statistics and Reproducibility” in the methods section.

Statistics and data presentation

Statistics: Wherever statistics have been derived (e.g. error bars, box plots, statistical significance) the legend needs to provide and define the n number (i.e. the sample size used to derive statistics) as a precise value (not a range), using the wording “n=X biologically independent samples/animals/cells/independent experiments/n= X cells examined over Y independent experiments” etc. as applicable.

The figure legends must indicate the statistical test used. Where appropriate, please indicate in the figure legends whether the statistical tests were one-sided or two-sided and whether adjustments were made for multiple comparisons.

- For null hypothesis testing, please indicate the test statistic (e.g. F, t, r) with confidence intervals, effect sizes, degrees of freedom and P values noted.
- Please provide the test results (e.g. P values) as exact values whenever possible and with confidence intervals noted.

Legends requiring revision:

1. Please indicate the statistical test used for data analysis and where appropriate, please specify whether it was one-sided or two-sided and whether adjustments were made for multiple comparisons, in the legends of **Figures 3b-e**.
2. Separately, in **Figure 3**, the sub-figure panel "d" is incorrectly labelled as "c". Please rectify the labelling, accordingly.

Please note that statistics such as error bars significance and p values cannot be derived from n<3 and must be removed in all such cases.

Data presentation: Please ensure that data presented in a plot, chart or other visual representation format shows data distribution clearly (e.g. dot plots, box-and-whisker plots). When using bar charts, please overlay the corresponding data points (as dot plots) whenever possible and always for n ≤ 10. (Please see the following editorial for the rationale behind this request and an example https://www.nature.com/articles/s41551-017-0079).

All error bars need to be defined in the legends (e.g. SD, SEM) together with a measure of centre (e.g. mean, median). For example, the legends should state something along the lines of “Data are presented as mean values +/- SEM” as appropriate.

All box plots need to be defined in the legends in terms of minima, maxima, centre, bounds of box and whiskers and percentile.

Other notes

We have included as an attachment to the decision letter a version of your Reporting Summary with a few notes. This is mainly for your information, but we hope it is helpful when preparing your revised manuscript. If you decide to resubmit the manuscript for further consideration, please be sure to include an updated Reporting Summary.
