# Supplementary material for: NetREX-CF integrates incomplete transcription factor data with gene expression to reconstruct gene regulatory networks
Source: Commun Biol. 2022 Nov 23;5:1282. doi: 10.1038/s42003-022-04226-7 (PMC9684490; doi:10.1038/s42003-022-04226-7)
Supplement: Supplementary file 3 — Supplementary Information [file 42003_2022_4226_MOESM3_ESM.pdf]

Supplementary Materials

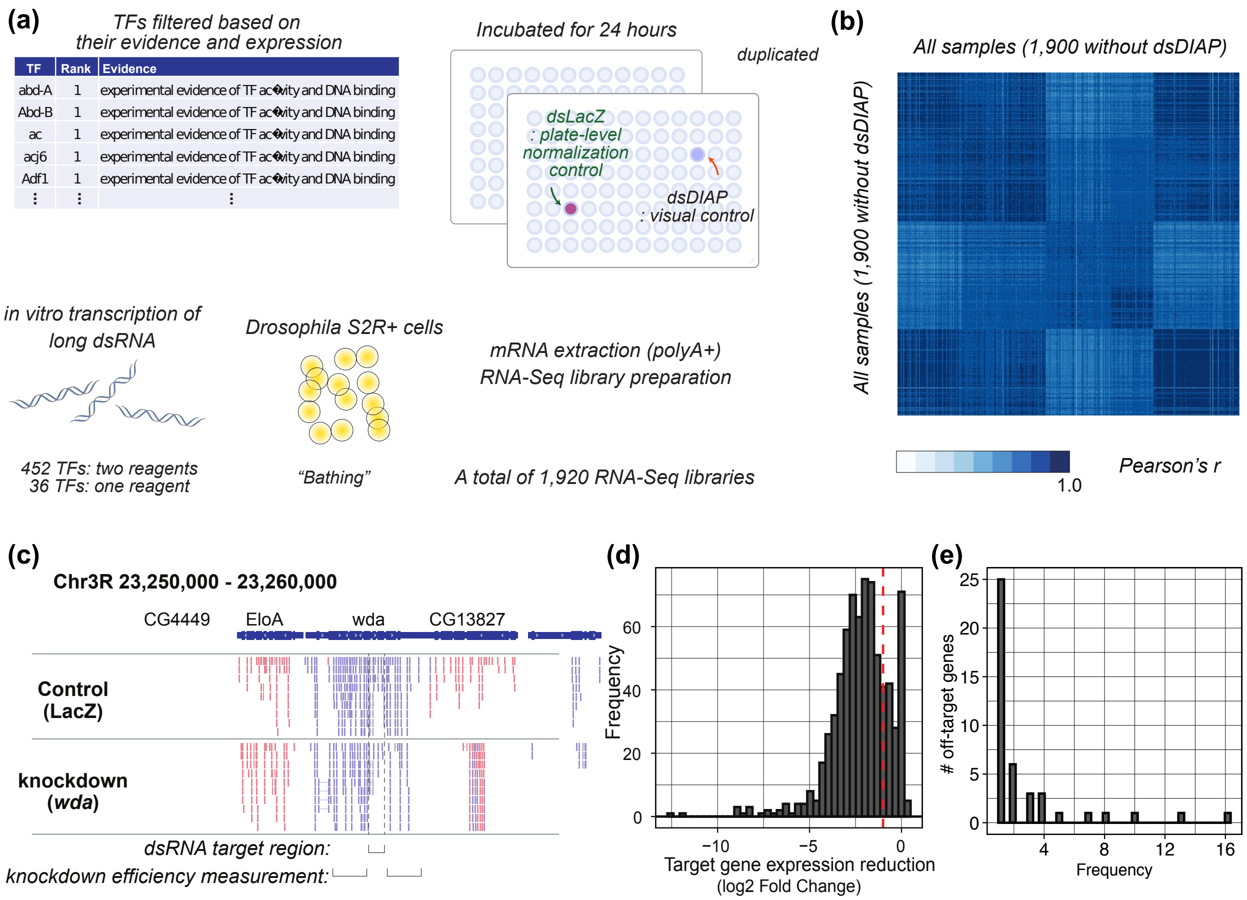

Supplementary Figure 1: A large-scale RNA-Seq analysis of *Drosophila* S2 cells with TF RNAi (a) A schematic illustration of the experimental design. dsLacZ. Cells treated with dsRNA against *E.coli* LacZ gene. dsDIAP. Cells treated with dsRNA against *Drosophila* Diap-1 gene (Death-associated inhibitor of apoptosis 1) (b) All sample-to-all sample pairwise correlation of gene expression is demonstrated as a heatmap. Color intensity corresponds to Pearson's correlation coefficient r. (c) An example of wda gene illustrates how short reads are mapped to the reference genome. Blue tiles. Reads that are aligned to 5' to 3' direction in the strand-specific RNA-Seq outcome. Red tiles. Reads for 3' to 5' direction. Top. The result from the control cells (*dsLacZ*). Bottom. Results from the cells treated with dsRNA for wda. Dot lines indicate the region for dsRNA targeting. Knockdown efficiencies were measured without reads mapped onto this region from both control and RNAi samples. (d) A histogram that shows the distribution of knockdown efficiency values, which are represented as reduction of target gene RNA (log2 Fold Changes between RNAi vs. Control). Dot red line. 50% reduction of mRNA (log2 Fold Change = - 1). (e) A histogram that shows an incident of significant off-target effects (adjusted p value ¡ 0.1) from all of the reagents (n = 941). We used BLAT [1] for the identification of any sequences that have more than 7-bp matching any of the reagents.

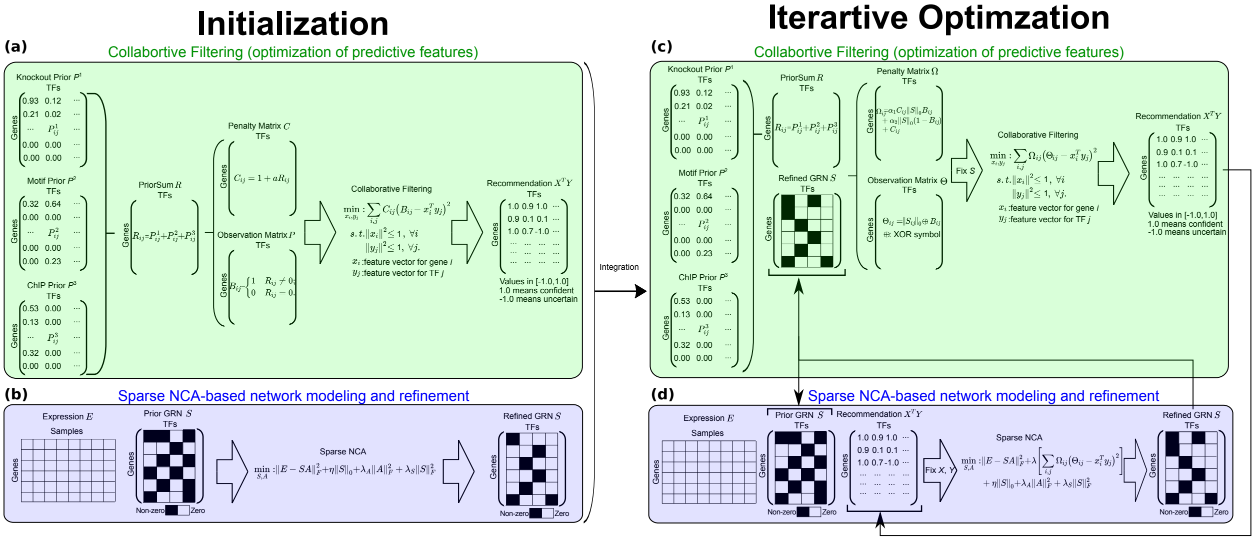

Supplementary Figure 2: The overview of the information flow in the NetREX-CF optimization.

Supplementary Note 1: GPALM Problem Introduction

We extend the original PALM algorithm [2] and propose the GPALM algorithm that can solve more general problems. The format of the problem that GPALM can solve is explained in this section. The actual algorithm and its convergence proof are provided in Supplementary Note 2.

The problem and basic assumptions

We are interested in solving the non-convex and non-smooth minimization problem with the following structure

$$\text{(M)} \qquad \min : \Psi\left(X, Y, Z\right) := H\left(X, Y\right) + F\left(Y, Z\right), \tag{1}$$

where we have the following assumption:

Assumption 1. *The assumptions for problem (M) is as follow:*

1. $H : \mathbb{R}^n \times \mathbb{R}^m \to \mathbb{R}$ *is a* C^1 *function.*
2. $F : \mathbb{R}^m \times \mathbb{R}^l \to (-\infty + \infty]$ *is a proper and lower semicontinuous (PLS) function. And* $F(Y, Z)$ *has the following structure* $F(Y, Z) := \sum_{i=1}^{m} p_i(Z) g_i(Y_i) + Q(Z)$*, where* $p_i : \mathbb{R}^l \to \mathbb{R}$ *is Lipschitz continuous with moduli* $L_i(Z)$ *and* $p_i(Z) > 0,\ \forall i$ *and* $g_i : \mathbb{R} \to \mathbb{R}$ *is lower semicontinuous and* $\sup g_i(Y_i) < \lambda_i,\ \forall i$ *and* $Q : \mathbb{R}^l \to \mathbb{R}$ *is Lipschitz continuous with moduli* $L_Q(Z)$*.* $Y = [Y_1,\ ...,\ Y_i,\ ...,\ Y_m]$*.*
3. $\inf_{\mathbb{R}^n \times \mathbb{R}^m} H > -\infty$ *and* $\inf_{\mathbb{R}^m \times \mathbb{R}^l} F > -\infty$*.*
4. *For any* Y *the function* $X \to H(X, Y)$ *is* $C^{1,1}_{L_X(Y)}$*, namely the partial gradient* $\nabla_X H(X, Y)$ *is globally Lipschitz with moduli* $L_1(Y)$*, that is*

   $$\left\| \nabla_X H(X_1, Y) - \nabla_X H(X_2, Y) \right\| \leq L_1(Y) \left\| X_1 - X_2 \right\|. \tag{2}$$

   Likewise, for any fixed X *the function* $Y_i \to H(X, Y_i)$ *is assumed to be* $C^{1,1}_{L_{Y_i}(X)}$*.*
5. *For any fixed* Y *the function* $Z \to F(Y, Z)$ *is assumed to be* $C^{1,1}_{L_Z(Y)}$*.*
6. ∇H *is Lipschitz continuous on bounded subsets of* $\mathbb{R}^n \times \mathbb{R}^m$*. In other words, for each bounded subsets* $T_1 \times T_2$ *of* $\mathbb{R}^n \times \mathbb{R}^m$ *there exist* $M > 0$ *such that any* (X_1, Y_1) *and* (X_2, Y_2)*:*

   $$\left\| \left(\nabla_X H\left(X_1, Y_1\right) - \nabla_X H\left(X_2, Y_2\right), \nabla_Y H\left(X_1, Y_1\right) - \nabla_Y H\left(X_2, Y_2\right) \right) \right\| \leq M \left\| \left(X_1 - X_2, Y_1 - Y_2\right) \right\|. \tag{3}$$

Subdifferentials of nonconvex and nonsmooth functions

Definition 1. *Let $\sigma : \mathbb{R}^d \to (-\infty, +\infty]$ be a PLS function. For a given $x \in \mathrm{dom}\ \sigma$, the Frechet subdifferential of σ at x, written $\hat{\partial}\sigma(x)$, is the set of all vectors $u \in \mathbb{R}^d$ which satisfy*

$$\lim_{y \neq x} \inf_{y \to x} \frac{\sigma(y) - \sigma(x) - < u, y - x >}{\|y - x\|} \geq 0. \tag{4}$$

When $x \in \mathrm{dom}\ \sigma$, we set $\hat{\partial}\sigma(x) = \emptyset$.

Proposition 1. *$\partial(\lambda f(x)) = \lambda \partial f(x)$ for any $\lambda > 0$.*

The proposition can be proved based on Definition 1.

Proximal map

Let $\sigma : \mathbb{R}^d \to (-\infty, +\infty]$ be a PLS function. Given $x \in \mathbb{R}^d$ and $t > 0$, the proximal map associate to σ id defined by:

$$\mathrm{prox}_{\lambda}^{\sigma}(x) := \arg\min \left\{ \sigma(u) + \frac{\lambda}{2} \|u - x\|, u \in \mathbb{R}^d \right\} \tag{5}$$

The proximal map has the following important property (Lemma 3.2 in [2]).

Lemma 1. *Let $h : \mathbb{R}^d \to \mathbb{R}$ be a continuously differentiable function with gradient ∇h assumed L_h Lipschitz continuous and let $\sigma : \mathbb{R}^d \to (-\infty, +\infty]$ be a proper and lower semi-continuous function with $\inf_{\mathbb{R}^d} \sigma > -\infty$. Fix any $t > L_h$, then for any $u \in \mathrm{dom}\sigma$ and any $u^+ \in \mathbb{R}^d$ defined by*

$$u^+ \in \mathrm{prox}_t^{\sigma}\left(u - \frac{1}{t}\nabla h(u)\right), \tag{6}$$

we have

$$h(u^+) + \sigma(u^+) \leq h(u) + \sigma(u) - \frac{1}{2}(t - L_h)\left\|u^+ - u\right\|^2. \tag{7}$$

Supplementary Note 2: GPALM Algorithm and its Convergence Analysis

The Algorithm

Here we first write out the algorithm that is able to solve the problem (M) with a convergence guarantee.

Convergence analysis

The proof procedure has followed the proofs introduced in the original PALM algorithm [2].

Supplementary Table 1: The GPALM algorithm.

Algorithm 1: The algorithm for the problem (M).

Initialization: X^0, Y^0, and Z^0.

for $k = 0,\ 1,\ ...,\ K$ **do**

$$X^{k+1} \in \operatorname{prox}_{\mu_X^k}^{H\left(\cdot, Y^k\right)}\left(X^k\right) \tag{8}$$

for $i = 0,\ 1,\ ...,\ m$ **do**

$$Y_i^{k+1} \in \operatorname{prox}_{\mu_{Y_i}^k}^{F\left(\cdot, Z^k\right)}\left(Y_i^k - \frac{1}{\mu_{Y_i}^k}\nabla_{Y_i} H\left(X^{k+1}, Y_i^k\right)\right) \tag{9}$$

end

$$Z^{k+1} \in \operatorname{prox}_{\mu_Z^k}^{F\left(Y^{k+1}, \cdot\right)}\left(Z^k\right) \tag{10}$$

end

Theorem 1. *Assume $\Psi(B)$ is a PLS function with $\inf \Psi > -\infty$, the sequence $\left\{B^k\right\}_{k\in\mathbb{N}}$ is a Cauchy sequence and converges to a critical point of $\Psi(B)$, if the following four conditions hold [2]:*

(i) Sufficiently decreasing: there exist some positive constant $\rho_1 > 0$, such that

$$\Psi(B^k) - \Psi(B^{k+1}) \geq \rho_1 \left\|B^{k+1} - B^k\right\|^2, \forall k. \tag{11}$$

(ii) Relative error: there exist some positive constant $\rho_2 > 0$, such that for any $w^k \in \partial\Psi(B^k)$,

$$\left\|w^k\right\| \leq \rho_2 \left\|B^{k+1} - B^k\right\|, \forall k. \tag{12}$$

(iii) Continuity: there exist a subsequence $\left\{B^{k_j}\right\}_{j\in\mathbb{N}}$ and B^*, such that

$$B^{k_j} \to B^*,\ \Psi\left(B^{k_j}\right) \to \Psi\left(B^*\right),\ \text{as } j \to +\infty. \tag{13}$$

(iv) KL property: Ψ satisfies KL property in its effective domain.

By the theorem above, we only need to check that the sequence generated by Algorithm 6 satisfies the conditions (i) - (iv).

Proposition 2. *Algorithm 6 is a global convergence algorithm.*

Proof. Follow Theorem 1, we prove Algorithm 6 satisfies conditions (i)- (iv).

Condition (i). Based on (8), we know

$$X^{k+1} \in \operatorname{prox}_{\mu_X^k}^{H\left(\cdot, Y^k\right)}\left(X^k\right) = \arg\min\left\{H\left(X, Y^k\right) + \frac{1}{\mu_X^k}\left\|X - X^k\right\|, X \in \mathbb{R}^n\right\}, \tag{14}$$

which implies

$$H\left(X^{k+1},Y^{k}\right)+F\left(Y^{k},Z^{k}\right)\leq H\left(X^{k},Y^{k}\right)+F\left(Y^{k},Z^{k}\right)-\frac{\mu_{X}^{k}}{2}\left\|X^{k+1}-X^{k}\right\| \tag{15}$$

We then apply Lemma 1 to (9),

$$H\left(X^{k+1},Y_{i}^{k+1}\right)+F\left(Y_{i}^{k+1},Z^{k}\right)\leq H\left(X^{k+1},Y_{i}^{k}\right)+F\left(Y_{i}^{k},Z^{k}\right)-\frac{1}{2}\left(\mu_{Y_{i}}^{k}-L_{Y}(X^{k+1})\right)\left\|Y_{i}^{k+1}-Y_{i}^{k}\right\| \tag{16}$$

Similar to the derivation related to X, for Z we get

$$H\left(X^{k+1},Y^{k+1}\right)+F\left(Y^{k+1},Z^{k+1}\right)\leq H\left(X^{k+1},Y^{k+1}\right)+F\left(Y^{k+1},Z^{k}\right)-\frac{\mu_{Z}^{k}}{2}\left\|Z^{k+1}-Z^{k}\right\| \tag{17}$$

Let $B^{k}=\left(X^{k},Y^{k},Z^{k}\right)$ and sum over equations from (15) to (17). We have

$$\Psi\left(B^{k+1}\right)\leq\Psi\left(B^{k}\right)-\frac{\mu_{X}^{k}}{2}\left\|X^{k+1}-X^{k}\right\|-\sum_{i}\frac{1}{2}\left(\mu_{Y_{i}}^{k}-L_{Y_{i}}(X^{k+1})\right)\left\|Y_{i}^{k+1}-Y_{i}^{k}\right\|-\frac{\mu_{Z}^{k}}{2}\left\|Z^{k+1}-Z^{k}\right\|. \tag{18}$$

We know that μ_{X}^{k}, μ_{Y}^{k}, and μ_{Z}^{k} have their lower bound and $\mu_{Y_{i}}^{k}>L_{Y}(X^{k+1})$. Therefore, we can get $\rho_{1}=\frac{\mu_{Y}^{k}}{2}+\sum_{i}\frac{1}{2}\left(\mu_{Y_{i}}^{k}-L_{Y_{i}}(X^{k+1})\right)+\frac{\mu_{Z}^{k}}{2}$. Then for $B^{k}=\left(X^{k},Y^{k},Z^{k}\right)$ we have

$$\Psi(B^{k})-\Psi(B^{k+1})\geq\rho_{1}\left\|B^{k+1}-B^{k}\right\|^{2},\forall k \tag{19}$$

tha proves condition (i).

Condition (ii). Writing down the optimality condition for (8), we have

$$\nabla_{X}H\left(X^{k-1},Y^{k-1}\right)+\mu_{X}^{k-1}\left(X^{k}-X^{k-1}\right)=0. \tag{20}$$

Let $w_{X}^{k}:=-\mu_{X}^{k-1}\left(X^{k}-X^{k-1}\right)-\nabla_{X}H\left(X^{k-1},Y^{k-1}\right)+\nabla_{X}H\left(X^{k},Y^{k}\right)$. It is easy to prove that $w_{X}^{k}\in\partial_{X}\Psi\left(X^{k},Y^{k},Z^{k}\right)$. Then

$$\begin{aligned}\left\|w_{X}^{k}\right\|&\leq\mu_{X}^{k-1}\left\|X^{k}-X^{k-1}\right\|+\left\|\nabla_{X}H\left(X^{k},Y^{k}\right)-\nabla_{X}H\left(X^{k-1},Y^{k-1}\right)\right\|\\&\leq\mu_{X}^{k-1}\left\|X^{k}-X^{k-1}\right\|+M\left(\left\|X^{k}-X^{k-1}\right\|+\left\|Y^{k}-Y^{k-1}\right\|\right)\\&\leq\left(\mu_{X}^{k-1}+2M\right)\left\|B^{k}-B^{k-1}\right\|.\end{aligned} \tag{21}$$

The first inequality comes from the fact that ∇H is Lipschitz continuous on bounded subset $\mathbb{R}^{n}\times\mathbb{R}^{m}$ as assumed in Assumption 1 (6).
With the optimality condition for (9), we have

$$\nabla_{Y_{i}}H\left(X^{k},Y_{i}^{k-1}\right)+\mu_{Y_{i}}^{k-1}\left(Y_{i}^{k}-Y_{i}^{k-1}\right)+\partial_{Y_{i}}F\left(Y_{i}^{k},Z^{k-1}\right)=0. \tag{22}$$

Let $w_{Y_i}^k := -\mu_{Y_i}^k \left(Y_i^{k+1} - Y_i^k\right) - \nabla_{Y_i} H\left(X^k, Y_i^{k-1}\right) + \nabla_{Y_i} H\left(X^k, Y_i^k\right) - \partial_{Y_i} F\left(Y_i^k, Z^{k-1}\right) + \partial_{Y_i} F\left(Y_i^k, Z^k\right)$. Clearly, $w_{Y_i}^k \in \partial_Y \Psi\left(X^k, \cdots, Y_{i-1}^k, Y_i^k, Y_{i+1}^k, \cdots, Z^k\right)$, then we have

$$\begin{aligned}
\left\|w_{Y_i}^k\right\| &\leq \mu_{Y_i}^k \left\|Y_i^{k+1} - Y_i^k\right\| + \left\|\nabla_{Y_i} H\left(X^k, Y_i^k\right) - \nabla_{Y_i} H\left(X^k, Y_i^{k-1}\right)\right\| + \left\|\partial_{Y_i} F\left(Y_i^k, Z^k\right) - \partial_{Y_i} F\left(Y_i^k, Z^{k-1}\right)\right\| \\
&\leq \mu_{Y_i}^k \left\|Y_i^{k+1} - Y_i^k\right\| + M_{Y_i} \left\|Y_i^{k+1} - Y_i^k\right\| + \left\|\partial_{Y_i}\left(p_i(Z^k) g_i(Y_i)\right) - \partial_{Y_i}\left(p_i(Z^{k-1}) g_i(Y_i)\right)\right\| \\
&\leq \left(\mu_{Y_i}^k + M_{Y_i}\right) \left\|Y_i^{k+1} - Y_i^k\right\| + \left\|\partial g_i(Y_i)\left(p_i(Z^k) - p_i(Z^{k-1})\right)\right\| \\
&\leq \left(\mu_{Y_i}^k + M_{Y_i}\right) \left\|Y_i^{k+1} - Y_i^k\right\| + M_i^Z \left\|\partial g_i(Y_i)\right\| \left\|\left(Z^k - Z^{k-1}\right)\right\| \\
&\leq \left(\mu_{Y_i}^k + M_{Y_i} + M_i^Z U_{Y_i}\right) \left\|B^k - B^{k-1}\right\|.
\end{aligned} \tag{23}$$

The second inequality utilizes the structure of $F(Y, X)$ introduced in Assumption 1 (2). The third inequality uses Proposition 1. We set $M_{Y_i} > L_{Y_i}(X)$, $M_i^Z > L_i(Z)$, and $U_{Y_i} > U_i$. Similar to things related to X, writing down the optimality condition for (10),

$$\nabla_Z F\left(Y^k, Z^{k-1}\right) + \mu_Z^{k-1}\left(Z^k - Z^{k-1}\right) = 0. \tag{24}$$

Let $w_Z^k := -\mu_Z^{k-1}\left(Z^k - Z^{k-1}\right) - \nabla_Z F\left(Y^k, Z^{k-1}\right) + \nabla_Z F\left(Y^k, Z^k\right)$. We find that $w_Z^k \in \partial_Z \Psi\left(X^k, Y^k, Z^k\right)$ and we have

$$\left\|w_Z^k\right\| \leq \left(\mu_Z^{k-1} + M_Z\right) \left\|B^{k+1} - B^k\right\|, \tag{25}$$

where $M_Z > L_Z(Y)$.
Let $\rho_2 = \max\left\{\mu_X^{k-1} + 2M, \mu_{Y_i}^k + M_{Y_i} + M_i^Z U_{Y_i}, \mu_Z^{k-1} + M_Z\right\}$ and sum (21), (23), (25), we have

$$\left\|w^k\right\| \leq \rho_2 \left\|B^{k+1} - B^k\right\|, \tag{26}$$

where $w^k = \left(w_X^k, ..., w_Y^k, ..., w_Z^k\right) = \left(\partial_X^k \Psi, ..., \partial_{Y_1^k} \Psi, ..., \partial_{Z^k} \Psi\right) = \partial\Psi(X^k, Y^k, X^k) \in \partial\Psi(B^k)$.

Condition (iii). Summing (19) from $k = 0$ to $N - 1$ we have

$$\rho_1 \sum_k^{N-1} \left\|B^{k+1} - B^k\right\|^2 \leq \Psi(B^0) - \Psi(B^N) \tag{27}$$

Since $\left\{\Psi(B^N)\right\}$ is decreasing and $\inf \Psi > -\infty$, there exist some $\bar{\Psi}$ such that $\Psi(B^N) \to \bar{\Psi}$ as $N \to +\infty$. Therefore, let $N \to +\infty$ in (27), we have

$$\rho_1 \sum_k^{+\infty} \left\|B^{k+1} - B^k\right\|^2 \leq \Psi(B^0) - \bar{\Psi}, \tag{28}$$

which implies that $\lim \left\|B^k - B^{k-1}\right\| = 0$. Let $B^* = (X^*, Y^*, Z^*)$ be a limit point of $\left\{B^k\right\}_{k\in\mathbb{N}} = \left\{(X^k, Y^k, Z^k)\right\}_{k\in\mathbb{N}}$. Then (28) indicates that there is a subsequence $\left\{(X^{k_j}, Y^{k_j}, Z^{k_j})\right\}_{j\in\mathbb{N}}$ such that $(X^{k_j}, Y^{k_j}, Z^{k_j}) \to (X^*, Y^*, Z^*)$ as $j \to +\infty$.
From (9), we know

$$Y_i^{k+1} \in \arg\min\left\{< Y - Y_i^k, \nabla_{Y_i} H\left(X^k, Y_i^k\right) > + \frac{\mu_{Y_i}^k}{2} \left\|Y - Y_i^k\right\|^2 + F(Y, Z^k)\right\} \tag{29}$$

Let $Y = Y_i^*$ the limiting point of $\left\{Y_i^k\right\}_{k\in\mathbb{N}}$, we have

$$\begin{aligned} &< Y_i^{k+1} - Y_i^k, \nabla_{Y_i} H\left(X^k, Y_i^k\right) > + \frac{\mu_{Y_i}^k}{2}\left\|Y_i^{k+1} - Y_i^k\right\|^2 + F(Y_i^{k+1}, Z^k) \\ &\leq < Y_i^* - Y_i^k, \nabla_{Y_i} H\left(X^k, Y_i^k\right) > + \frac{\mu_{Y_i}^k}{2}\left\|Y_i^* - Y_i^k\right\|^2 + F(Y_i^*, Z^k) \end{aligned} \tag{30}$$

Set $k = k_j - 1$, we obtain

$$\begin{aligned} &< Y_i^{k_j} - Y_i^{k_j-1}, \nabla_{Y_i} H\left(X^{k_j-1}, Y_i^{k_j-1}\right) > + \frac{\mu_{Y_i}^{k_j-1}}{2}\left\|Y_i^{k_j} - Y_i^{k_j-1}\right\|^2 + F(Y_i^{k_j}, Z^{k_j-1}) \\ &\leq < Y_i^* - Y_i^{k_j-1}, \nabla_{Y_i} H\left(X^{k_j-1}, Y_i^{k_j-1}\right) > + \frac{\mu_{Y_i}^{k_j-1}}{2}\left\|Y_i^* - Y_i^{k_j-1}\right\|^2 + F(Y_i^*, Z^{k_j-1}) \end{aligned} \tag{31}$$

Let $j \to +\infty$, we get

$$\lim_{j\to+\infty} \sup F(Y_i^{k_j}, Z^{k_j-1}) \leq F(Y_i^*, Z^*) \tag{32}$$

From the fact that F is a PLS function, we also have

$$\lim_{j\to+\infty} \sup F(Y_i^{k_j}, Z^{k_j-1}) \geq F(Y_i^*, Z^*) \tag{33}$$

Based on (32) and (33), we know $\lim_{j\to+\infty} = F(Y_i^*, Z^*)$. Arguing similarly with X, we finally have

$$\lim_{j\to+\infty} \Psi(X^{k_j}, Y^{k_j}, Z^{k_j}) = \lim_{j\to+\infty} H(X^{k_j}, Y^{k_j}) + F(Y^{k_j}, Z^{k_j}) = \Psi(X^*, Y^*, Z^*). \tag{34}$$

Condition (iv). The function Ψ is a semi-algebraic function, which automatically satisfies the Kurdyka-Lojasiewicz property [2]. □

Supplementary Note 3: Parameter Selection for Algorithms used in the study

In this section, we introduce how we select parameters for the competing algorithms.

Paramter Selection for PriroSum

PriorSum constructs a predicted GRN by summing overweights from all prior networks $P = \{P^1, ..., P^d\}$. Therefore, PriorSum builds a GRN $\mathfrak{P}_{ij} = \sum_k P_{ij}^k$ and does not need to select any parameters.

Parameter Selection for LassoStARS

LassoStARS [3] is the latest version of Inferelator, it takes an unweighted prior and gene expression data as input. Because LassoStARS needs an unweighted prior network and the prior networks we have are weighted prior networks, we choose different cutoffs to construct prior networks for LassoStARS. We generate prior networks by assigning each gene the top N TFs based on the $\mathfrak{P}_{ij}$. For N, we set $N = \{10, 20, 30, 40\}$ and we find that $N = 10$ performs the best and report the results in the main paper. For other parameters used in LassoStARS, LassoStARS proposed a way to select the optimal parameters, therefore, we do not need to select other parameters.

Parameter Selection for MerlinP

For reconstructing the GRN for yeast, MerlinP [4] uses the same prior networks and gene expression to build a GRN and reported in the repository `https://github.com/Roy-lab/merlin-p`. We directly download the GRN they build and compared it with other methods. For reconstructing the S2 cell GRN and cell-specific GRNs, we follow the instruction provided in `https://github.com/Roy-lab/merlin-p`.

Parameter Selection for NetREX

NetREX [5] is similar to LassoStARS, taking an unweighted prior and gene expression as input. So similarly, we generate prior networks for NetREX by assigning each gene the top N TFs based on the $\mathfrak{P}_{ij}$. We set $N = \{10, 20, 30, 40\}$ and we find that $N = 20$ performs the best and report the results in the main paper. For the other parameters, we selected based on the suggestion provided in `https://github.com/ncbi/NetREX`.

Parameter Selection for CF

We input CF [6] with $\mathfrak{P}_{ij} = \sum_k P^k_{ij}$. The dimension of the hidden feature vector we set it to be 100, 200, and 300. The regulation term used by CF is set to be 0.1, 1, 10, 100. We try all those combinations and report the result with the best performance.

Parameter Selection for NetREX-CF

Based on the formulaiton of NetREX-CF (**??**), we know that we need to select h, λ_A, λ_S, η_{ij}, λ, and $\bar{C}_{ij}$. h is the dimension of the hidden feature vector. We find that $h = \{100, 200, 300\}$ does not change the performance much. For computational consideration, we set $h = 100$. Because λ_A and λ_S are used as standard regulation to avoid over-fitting, we set $\lambda_A = 1.0$ and $\lambda_S = 1.0$ by default. We introduce the selection of η_{ij} and $\bar{C}_{ij}$ in the following subsection.

Selection of η_{ij}

We need to make sure $F(S,X,Y)$ is lower semi-continuous. We can first simplify the equation into

$$
\begin{aligned}
F(S,X,Y) &= \lambda\left[\sum_{i,j}\Omega_{ij}\left(\|S_{ij}\|_0 + (1-\|S_{ij}\|_0)B_{ij} - x_i^T y_j\right)^2\right] + \sum_{i,j}\eta_{ij}\|S_{ij}\|_0 \\
&= \lambda\left[\sum_{i,j}(C_{ij} + (\bar{C}_{ij} - C_{ij})\|S_{ij}\|_0)\left(\|S_{ij}\|_0 + (1-\|S_{ij}\|_0)B_{ij} - x_i^T y_j\right)^2\right] + \sum_{i,j}\eta_{ij}\|S_{ij}\|_0 \\
&= \sum_{i,j}\left\{\lambda\left[\bar{C}_{ij}(1-B_{ij})(1+2(B_{ij}-x_i^T y_j)) + (\bar{C}_{ij}-C_{ij})(B_{ij}-x_i^T y_j)^2\right] + \ \eta_{ij}\right\}\|S_{ij}\|_0 \\
&\quad + \sum_{ij} C_{ij}(B_{ij} - x_i^T y_j)^2
\end{aligned}
\tag{35}
$$

$F(S,X,Y)$ is lower semi-continuous when the parameter before $\|S_{ij}\|_0$ in the above equation is larger than 0. After several manipulations, we find out we need to set η_{ij} as follows to make $F(S,X,Y$ lower semi-continuous.

$$
\eta_{ij} = \begin{cases} \geq 0, & B_{ij} = 1, \\ \geq \lambda\frac{C_{ij}\bar{C}_{ij}}{\bar{C}_{ij}-C_{ij}}, & B_{ij} = 0. \end{cases}
\tag{36}
$$

Selection of $\bar{C}_{ij}$

C_{ij} is the penalty when we want to use $x_i^T y_j$ to learn $B_{ij} = 1$. Similarly, $\bar{C}_{ij}$ is the penalty when we want to use $x_i^T y_j$ to learn $\|S_{ij}\|_0 = 1$. There are two siutations. First, when $\|S_{ij}\|_0 = 1$ and $B_{ij} = 1$, meaning the sparse NCA-based method confirms the edge in the prior, then intuitively, we need to set $\bar{C}_{ij} = \alpha C_{ij}, \alpha \geq 1$. Another situation is that $\|S_{ij}\|_0 = 1$ and $B_{ij} = 0$, meaning the sparse NCA-based model confirms an edges recommended by the CF model but not appeared in the prior networks. For this case, we set $\bar{C}_{ij} \in [C_{ij}, \max(C)]$, where $\max(C)$ is the largest element in penalty matrix C. In sum, $\bar{C}_{ij} = \alpha C_{ij}\|S_{ij}\|_0 B_{ij} + \beta\|S_{ij}\|_0(1-B_{ij})$, where $\alpha \geq 1$ and $\beta \in [C_{ij}, \max(C)]$.

Consensus of Different Parameter Selections

As explained in the previous, for η_{ij} and $\bar{C}_{ij}$, we know the range of these parameters but do not know the exact optimal values. For reconstructing GRN for the yeast experiment, we set

$$
\eta_{ij} = \begin{cases} \geq \theta, & B_{ij} = 1, \\ \geq \lambda\frac{C_{ij}\bar{C}_{ij}}{\bar{C}_{ij}-C_{ij}} + \theta, & B_{ij} = 0, \end{cases}
\tag{37}
$$

where $\theta = \{0.1, 0.5, 1, 2\}$. And $\bar{C}_{ij} = \alpha C_{ij}\|S_{ij}\|_0 B_{ij} + \beta\|S_{ij}\|_0(1-B_{ij})$, where $\alpha = \{1,2,3,10\}$ and $\beta = 10, 20, 30, 40$. For different set of parameters, we get a GRN and we get a set of GRNs $\mathfrak{G} = \{G^1, ...\}$, where $G^i = X^T Y$ after applying all theses parameters. The final perdition is the average overall predictions $G^* = \dfrac{\sum_i G^i}{|\mathfrak{G}|}$.

Supplementary Note 4: Data processing

ScRNA-Seq data

For all scRNA-Seq data used in the manuscript, to reduce the impact of the sparsity of the scRNA-Seq data, we eliminated cells with less than 500 genes expressed and genes that are expressed in fewer than 10% of the cells.

Supplementary References

[1] W. J. Kent. "BLAT–the BLAST-like alignment tool". In: *Genome Res* 12.4 (Apr. 2002), pp. 656–664.

[2] Jérôme Bolte, Shoham Sabach, and Marc Teboulle. "Proximal alternating linearized minimization for nonconvex and nonsmooth problems". In: *Mathematical Programming* 146.1-2 (2014), pp. 459–494.

[3] E. R. Miraldi et al. "Leveraging chromatin accessibility for transcriptional regulatory network inference in T Helper 17 Cells". In: *Genome Res.* 29.3 (Mar. 2019), pp. 449–463.

[4] Alireza F Siahpirani and Sushmita Roy. "A prior-based integrative framework for functional transcriptional regulatory network inference." In: *Nucleic acids research* 45.4 (2016), gkw963.

[5] Y. Wang et al. "Reprogramming of regulatory network using expression uncovers sex-specific gene regulation in Drosophila". In: *Nat Commun* 9.1 (Oct. 2018), p. 4061.

[6] Y. Hu, Y. Koren, and C. Volinsky. "Collaborative filtering for implicit feedback datasets". In: *Data Mining, 2008. ICDM'08. Eighth IEEE International Conference on.* IEEE. 2008, pp. 263–272.
