# Supplementary material for: NetREX-CF integrates incomplete transcription factor data with gene expression to reconstruct gene regulatory networks
Source: Commun Biol. 2022 Nov 23;5:1282. doi: 10.1038/s42003-022-04226-7 (PMC9684490; doi:10.1038/s42003-022-04226-7)
Supplement: Supplementary file 4 — Description of Additional Supplementary Files [file 42003_2022_4226_MOESM4_ESM.pdf]

Description of Additional Supplementary Files

File: Supplementary Data 1

Description: List of TFs and Genes information in the RNAi experiments.

File: Supplementary Data 2

Description: SRA ids of collected TF ChIP-seq data.
